# On the central mechanisms of normalizing effect of vagus nerve stimulation

**DOI:** 10.1192/j.eurpsy.2025.300

**Published:** 2025-08-26

**Authors:** S. Tukaiev, N. Vysokov, Y. Gachshenko, D. Toleukhanov, I. Brak, G. Datkhabayeva, O. Korbush, S. Danylov, V. Komarenko, J. M. A. Ferreira, M. Makarchuk

**Affiliations:** 1 Taras Shevchenko National University of Kyiv, Kyiv, Ukraine; 2 BrainPatch Ltd, Dublin, Ireland; 3 Cognitech Ltd, Astana; 4 Al-Farabi Kazakh National University, Almaty, Kazakhstan; 5 National University of Ukraine on Physical Education and Sport; 6 Beehiveor Academy and R&D Labs, Kyiv, Ukraine; 7 Universidade de Coimbra, Coimbra, Portugal

## Abstract

**Introduction:**

Neuromodulation technologies became the safe and effective alternative to treat psychiatric and neurological disorders. The current evidence from the up-to-date state in vagus nerve stimulation application in studies of emotional state modulation demonstrates a strong impact of vagus stimulation on the state of patients with a range of disorders. The normalizing effect of vagus nerve stimulation on abnormal sympathetic nerve activity may explain the beneficial effect on burnout, which is thought to be a manifestation of decreased vagus-mediated heart rate variability (HRV) under everyday stress.

**Objectives:**

Considering all of the above, we aimed to explore the influence of a novel VNS stimulation protocol on emotional state, as well as highlighting specific features of the activation-related neurodynamics and the effects on HRV time, frequency and non-linear metrics.

**Methods:**

11 right-handed male volunteers aged 18-22 years participated in the 1st (EEG) study (6 persons-taVNS group and 5-SHAM) and 62 healthy volunteers 18-49 years old were recruited for 2nd (HRV) study (26-VNS group and 22–Sham). We used the combination of pleasant meditative classical music and a slow bi-polar wave of electrical non-invasive transcutaneous stimulation of auricular area (BrainPatch platform for non-invasive stimulation). Vagus nerve stimulation was arranged as a 4-day course. Psychological testing (State Anxiety, STAI; psychological stress level, PSM-25; severity of emotional burnout, MBI; depression, IDS) was carried out.

**Results:**

Non-invasive stimulation was rated by the participants as a positive experience. We detected beneficial changes in the psychoemotional state of the respondents (improvement of mood, reduction of job related stress – emotional burnout). The increase of vagally mediated parameter RMSSD and decrease of LF/HF ratio has reflected the activation of parasympathetic nervous system (PNS) during stimulation. HRV effects of VNS turned out to be short-term, which was manifested in a drop in the value immediately after the stimulation. EEG analysis indicated a long-term effect of VNS. Increased alpha and beta rhythms (generalized growth in the frontal and posterior cortex) and gamma activity (frontal region) after a series of 4 VNS sessions may indicate the improvement of mental processes and creative thinking (attention, information processing and memory storage). Enhanced activation level was mirrored in an increased beta/alpha ratio.

**Conclusions:**

Our data suggests that VNS has a normalizing effect on the psychoemotional state shifting “sympatho-vagal balance” to the functional optimum. EEG data showed the prolonged stimulating effect on the brain processes related to the cognitive functioning while attenuating the stress impacts at the same time.

**Disclosure of Interest:**

None Declared

